# A new approach to assessment of the left ventricle

**DOI:** 10.1016/j.mex.2016.03.014

**Published:** 2016-03-29

**Authors:** Katrina K. Poppe, Rob N. Doughty, Gillian A. Whalley, Christopher M. Triggs

**Affiliations:** a Department of Medicine, University of Auckland, Auckland, New Zealand; b Department of Statistics, University of Auckland, Auckland, New Zealand

**Keywords:** Functional data analysis, functional data analysis, dynamics, kinematics, left ventricle, sub-clinical dysfunction, imaging, echocardiography

## Abstract

Cardiac motion is a continuous process; however most measurements to assess cardiac function are taken at brief moments in the cardiac cycle. Using functional data analysis, repeated measurements of left ventricular volume recorded at each frame of a continuous image measured with cardiac ultrasound (echocardiography) were turned into a function of volume over time. The first derivative of the displacement of volume with respect to time is velocity; the second derivative is acceleration. Plotting volume, velocity, and acceleration against each other in a 3-dimensional plot results in a closed loop. The area within the loop is defined by the kinematics of volume change and so may represent ventricular function.

• We have developed an approach to analyzing images of the left ventricle that incorporates information from throughout the cardiac cycle.

• Comparing systolic and diastolic areas within a loop defined by volume, velocity, and acceleration of left ventricular volume highlights imbalances in the kinematics of the two phases, potentially indicating early sub-clinical disease.
• Substantially more information about left ventricular function may be derived from a non-invasive clinically available tool such as echocardiography.

## Method details

### Rationale

Cardiac motion is a continuous process; however most measurements to assess cardiac function are taken at brief moments in the cardiac cycle. Systole (contraction) and diastole (relaxation) are interrelated yet they are rarely considered collectively, oversimplifying the interplay of forces through the cardiac cycle. In clinical practice, the contractile function of the main pumping chamber of the heart, the left ventricle (LV), is typically quantified by the ejection fraction (EF), which is calculated from LV volumes at the two brief moments of end-systole and end-diastole. But it provides no information about the motion or duration of the systolic phase itself. All of the current clinically available non-invasive measures of cardiac function extrapolate overall performance from brief moments in time only, and do not incorporate the full phases of systole and diastole.

The LV exhibits periodic motion, with each cardiac cycle starting with the same blood volume at which the previous cycle ended. The net effect of ventricular contraction is displacement of blood, thus LV volumetric changes may be used as a surrogate for global LV motion.

The endocardial border of the LV can be traced and tracked throughout the cardiac cycle. Existing software estimates the volume contained within the endocardial border. This volume can be extracted for each frame of the continuous image, resulting in a dataset of volume measurements every 3-6 milliseconds (depending on the acquisition frame rate). Functional data analysis (FDA) replaces the repeated measurements at discrete moments in time with a function of that measurement over continuous time [1]. The first derivative of the displacement of volume with respect to time is velocity, or the rate of change in volume. The second derivative of displacement with respect to time is acceleration. This represents the addition or removal of forces that produce the change in volume. If the rate of change in volume displacement (velocity) is of interest then it follows that what causes that change (acceleration) is also of interest.

In mechanical systems, plotting volume against one or more of its derivatives creates a plot in ‘phase space’ − a space that represents a measurement and the rates of change of that measurement [1], [2]. Plotting the volume data with either method results in a closed loop because the motion of the heart is a periodic process. The area within the loop is defined by the kinematics of volume change and so may represent ventricular function. Zero velocity defines the border between systole and diastole, allowing areas to be calculated separately for the two phases under the same loading conditions.

Force-frequency studies on tissue samples from failing ventricles have found that along with a decrease in systolic force, diastolic force increases as the rate of stimulation increases [3], [4]. Cellular transport mechanisms become impaired in failing myocardium [4], [5] and chronic diabetes [6]. As a result of slowed decay of the intracellular calcium transient there is a deleterious accumulation of intracellular calcium, which is associated with a rise in diastolic force. The rise in force is from persistent activation of contractile proteins during diastole, requiring greater energy at rest and as rate increases. Comparing the systolic to diastolic area in our loops would highlight imbalances in the kinematics of the two phases, potentially indicating early sub-clinical disease, where early changes to myocardial function due to impaired glucose metabolism, for example, are thought to affect the diastolic phase before the systolic.

Thus the relationship between systolic and diastolic kinematics derived from a non-invasive clinically available tool such as echocardiography, may tap into findings that have previously only been able to be assessed in explanted tissue samples.

## Method

1. The volume data are replaced by a sum of periodic Fourier components. This sum specifies the form of the volume curve over time as a function. Numerical approximations to the first and second order derivatives are then calculated, representing velocity and acceleration respectively.
2. The dataset of time, volume, velocity and acceleration are each centred on their means so that the loop lies close to the projected plane, decreasing the risk of overestimation.
3. The interplay of the three dynamic dimensions is investigated using two-dimensional (2D) and 3D plots. The 2D plots compare volume against velocity, and acceleration against velocity. The 3D plot contains volume, velocity and acceleration.
4. Area is calculated using a method first described by Meister in 1769 [7] where, for a simple polygon with *n* vertices where vertex *i* has coordinates (*x_i_*, *y_i_*):
  $$Area=\frac{1}{2}\sum_{i=0}^{n-1}\left(x_{i}y_{i+1}-x_{i+1}y_{i}\right)$$
  A vertex is defined every millisecond, *x* is velocity, and *y* is volume or acceleration depending on the plot.
5. Zero velocity defines the border between systole and diastole allowing area to be calculated separately for the two phases. Thus the area defined by each phase is enclosed by specifying the first and last vertices to be the same, ie. $x_{n}y_{n}=x_{0}y_{0}$. This can be visualised as two arcs that hinge at the boundary between systole and diastole.
6. Each of the two phases is rotated through an orthogonal rotation matrix such that:
  i. Fixed starting points (*a, b, c, d*) on the curve are selected so that line segments *ab* and *cd* lie in the same plane, have unit length, and are orthogonal to each other.
  ii. A third vector is created that lies in the same plane, has unit length, and is orthogonal to *ab* and *cd*.
  iii. The curve in three dimensions is rotated to find the plane (in two dimensions) such that the projection of the curve on the plane has maximal area. We can think of the plane as being the best 2D approximation to the 3D curve.
7. The rotated curve is closed to allow calculation of area using the method described by Meister [6].
8. Area is optimised using the Nelder-Mead search algorithm [8]. As the curves are projected on to two dimensions, the algorithm works by directing area to be calculated at three slightly different angles or positions in the plane, represented as the three vertices of a triangle (a 2-simplex). The behaviour of the function calculating area at each test point is extrapolated and a new test position is generated. If the area at that point is larger than those at the previous points, the triangle ‘flips’ around the new point and area is measured at the new positions. This process continues until the maximum area is found.
9. The areas in each phase are calculated separately however the two curves are constrained to remain joined using a penalty term based on the distance between the start of systole and end of diastole, and the end of systole and start of diastole.
10. Systolic and diastolic areas are calculated. The absolute value of the areas is affected by physiological and pathological changes in ventricular size and does not allow direct comparison between patients. Therefore, the ratio of systolic to diastolic area is generated to create an internally standardised index of LV function, which we have termed the kinematic index (k-index).

### Method validation

**Fig. 1:**
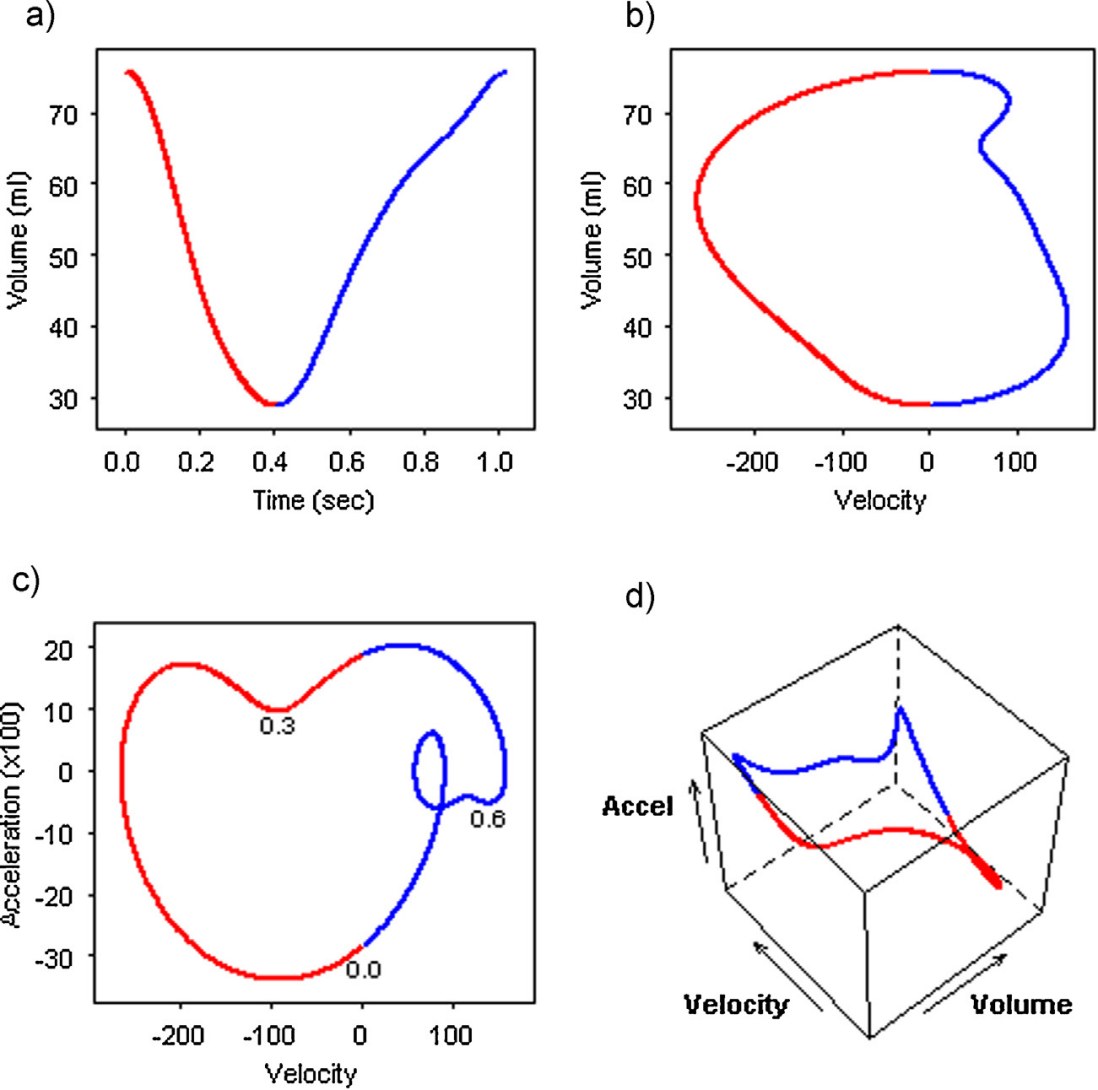
Kinematic plots of left ventricular volume. Data from one healthy volunteer a) fitted volume against time, b) volume against velocity, c) acceleration against velocity, d) volume against velocity against acceleration. Red = systole, blue = diastole.

The method was applied to images from 15 volunteers without high blood pressure, diabetes, or evidence of cardiovascular disease (age 19-42 years, 40% female, heart rate 54-91 bpm). The results from one individual are illustrated in Fig. 1.

The repeated measurements of LV volume measured from real-time 3D echocardiography through one cardiac cycle creates a plot of volume against time (Fig. 1a). The function was fitted to this curve, and the optimal fit obtained with a sum of nine Fourier basis functions comprising four pairs of sine and cosine waveforms.

The 2D plot of volume against velocity evolved a smooth loop without involutions (Fig. 1b). The left half of the loop (negative velocity) represents mechanical systole, and the right half (positive velocity) represents mechanical diastole. In the plot of acceleration against velocity (Fig. 1c), the intersection of zero velocity and maximal negative acceleration during the isovolumic phase was taken as the start of the cardiac cycle (time 0.0), before moving in a clockwise direction. Similarly, diastole started at zero velocity and maximal positive acceleration.

In the systolic phase, acceleration decreased as velocity increased through early systole until maximum negative systolic velocity was reached. Acceleration then increased with little change in velocity before a sustained slowing in rate and a reduction in acceleration toward a nadir one third of the way through the cycle, which may correspond to the end of the active ejection phase (time 0.3). The rate of ejection continued to slow however acceleration increased again until reaching its maximum during the isovolumic phase at end-systole. The systolic phase typically ended before half of the cardiac cycle had passed and had a fairly consistent appearance across all subjects.

During mechanical diastole, acceleration gradually decreased as velocity increased through early diastole before reaching maximum positive velocity. A sub-loop was typically seen between 0.6 and the end of the cardiac cycle, however there was greater variability in the appearance of the diastolic phases across subjects.

The 3D plot of volume against velocity against acceleration evolved a more complex loop, revealing the interplay between the three dynamic components in a way that could not be captured in the 2D plots (Fig. 1d). The loop followed a right angled or even sigmoid path and so a portion will always be perpendicular to the aspect presented in either one of the 2D plots.
